# *In vitro* and *in vivo* evaluation of a single chain antibody fragment generated *in planta* with potent rabies neutralisation activity

**DOI:** 10.1016/j.vaccine.2018.02.057

**Published:** 2019-08-02

**Authors:** Waranyoo Phoolcharoen, Ashley C. Banyard, Christophe Prehaud, David Selden, Guanghui Wu, Colin P.D. Birch, Tim H. Szeto, Monique Lafon, Anthony R. Fooks, Julian K.-C. Ma

**Affiliations:** a Institute for Infection and Immunity, St. George’s Hospital Medical School, University of London, London, UK; b Pharmacognosy and Pharmaceutical Botany, Faculty of Pharmaceutical Sciences, Chulalongkorn University, Bangkok, Thailand; c Wildlife Zoonoses and Vector-borne Diseases Research Group, Animal and Plant Health Agency (APHA), Addlestone, Surrey KT15 3NB, UK; d Institut Pasteur, Unité de Neuroimmunologie Virale, Département de Virologie, Paris, France; e Biomathematics and Risk Research Group, Animal and Plant Health Agency (APHA), Addlestone, Surrey KT15 3NB, UK

**Keywords:** Rabies virus, Single-chain antibody (ScFv), Blood brain barrier (BBB), Clinical disease, Immunoglobulin, N-acetylcholine receptor, *Nicotiana benthamiana*

## Abstract

Rabies causes more than 60,000 human deaths annually in areas where the virus is endemic. Importantly, rabies is one of the few pathogens for which there is no treatment following the onset of clinical disease with the outcome of infection being death in almost 100% of cases. Whilst vaccination, and the combination of vaccine and rabies immunoglobulin treatment for post-exposure administration are available, no tools have been identified that can reduce or prevent rabies virus replication once clinical disease has initiated. The search for effective antiviral molecules to treat those that have already developed clinical disease associated with rabies virus infection is considered one of the most important goals in rabies research. The current study assesses a single chain antibody molecule (ScFv) based on a monoclonal antibody that potently neutralises rabies *in vitro* as a potential therapeutic candidate. The recombinant ScFv was generated in *Nicotiana benthamiana* by transient expression, and was chemically conjugated (ScFv/RVG) to a 29 amino acid peptide, specific for nicotinic acetylcholine receptor (nAchR) binding in the CNS. This conjugated molecule was able to bind nAchR *in vitro* and enter neuronal cells more efficiently than ScFv. The ability of the ScFv/RVG to neutralise virus *in vivo* was assessed using a staggered administration where the molecule was inoculated either four hours before, two days after or four days after infection. The ScFv/RVG conjugate was evaluated in direct comparison with HRIG and a potential antiviral molecule, Favipiravir (also known as T-705) to indicate whether there was greater bioavailability of the ScFv in the brains of treated mice. The study indicated that the approach taken with the ScFv/RVG conjugate may have utility in the design and implementation of novel tools targetting rabies virus infection in the brain.

## 1 Introduction

Rabies is a neglected disease caused by Rabies virus (RABV) that affects people in many countries, mostly in Asia and Africa. RABV is a non-segmented negative strand RNA virus in the order *Mononegavirales*, family *Rhabdoviridae*, genus *lyssavirus* [1]. Rhabdoviruses are enveloped with a typical bullet- or rod-shaped morphology and characterized by an extremely broad host spectrum ranging from plants to insects to mammals. The genome encodes five proteins including nucleoprotein, phosphoprotein, matrix protein, glycoprotein, and RNA polymerase.

RABV is almost always transmitted following a bite injury from an infected animal that is excreting virus in its saliva. The mechanism of virus infection once it has crossed the dermal barrier is poorly defined. Lyssaviruses are strongly neurotrophic, however, replication in the musculature, prior to entry into the peripheral nervous system occurs, and is likely to contribute to the variation in incubation times seen following infection [2]. Whilst the prodrome generally lasts for 3–10 weeks, significantly longer incubation periods have been reported [3]. Regardless, it is during the phase between virus replication in the non-neuronal periphery and movement into the peripheral nervous system that post exposure immunoprophylaxis is hypothesised to be most effective [4].

Current options for rabies post exposure treatment include immunoprohylaxis with human or equine rabies immunoglobulin (H/ERIG) at the site of the infection and vaccination at a site distant from the exposure to ensure that the application of RIG does not interfere with the humoral immune response [5]. Rabies post-exposure prophylaxis (PEP) is highly effective if administered in a timely manner following exposure [6], [7], [8], [9]. However, in endemic regions, knowledge of the most effective actions to take following an exposure event is often limited, as is the availability of PEP. Furthermore, in remote areas, travel to medical centres for treatment can delay treatment. If clinical disease develops, PEP is entirely ineffective [10], [11], [12]. Rabies virus antibodies, such as RIG are unlikely to offer therapeutic benefits once rabies virus (RABV) has entered the CNS, as they cannot cross the blood brain barrier (BBB), a dense cellular network that extends along all capillaries and consists of tight junctions of endothelial cells that prevent the entry of large bacterial pathogens and molecules into the cerebrospinal fluid. The size exclusion limit is approximately 10 kD [13].

Rabies glycoprotein (G), present as a trimeric peplomer on the viral envelope, contains a short conserved motif which serves to bind cellular receptors [14], including nicotinic acetylcholine receptors (nAchRs), to mediate entry into cells [15]. Prior to the establishment of a productive infection of the CNS, RABV utilises nAchRs [16] to enter both muscle and nerve cells in the periphery [17], [18], [19], [20]. The identification of a key 29 amino acid peptide in G responsible for binding and entry into neuronal cells led to the demonstration that other molecules (siRNA) [21], nanoparticles [22], [23], and enzymes [24], [25]] could be delivered to the CNS if linked to this peptide.

Previous studies have described the application of monoclonal antibody preparations as an alternative to RIG [26], generation of monoclonal antibodies *in planta* and expression of a single chain antibody fragment (ScFv) of a previously defined rabies neutralising monoclonal antibody in *E. coli* [27] and *N. benthamiana* [28]. In the latter study, a fusion protein comprising ScFv linked to the RVG peptide at its C-terminus was expressed and shown to neutralise RABV, bind to nAchR and transport across a model BBB. However, ScFv-RVG fusion was poorly expressed, so although promising, this strategy was not deemed feasible for further development. In the current study, the ScFv was expressed also in *N. benthamiana* but chemically conjugated to synthetic 29 amino acid peptide (ScFv/RVG) for evaluation. The ScFv/RVG conjugate retained the ability to neutralise RABV. In comparison to ScFv alone, ScFv/RVG demonstrated enhanced ability to cross an *in vivo* 3D cell culture BBB model via a mechanism that involves the N-acetylcholine receptor. Finally, the ability of ScFv/RVG to act as a potential post-exposure tool was assessed *in vivo*. Direct *in vivo* comparisons with the action of HRIG demonstrated that ScFv/RVG may have future utility as a post-exposure alternative to HRIG for rabies virus post exposure treatment.

## 2 Materials and methods

### 2.1 ScFv and ScFv/RVG production

The pEAQ-62-71-3 IgG [27] and the pEAQ-ScFv vectors used for expression of recombinant antibodies have been described previously [29]. *Agrobacterium tumefaciens* LBA4404 was separately transformed with the pEAQ-62-71-3 IgG [27] and the pEAQ-ScFV [28] vectors by electroporation. The resulting recombinant bacterial strains were verified by restriction digest of plasmids, grown overnight at 28 °C and used to infiltrate leaves of 6–8 week-old greenhouse-grown *N. benthamiana* plants, by vacuum infiltration as described [30]. The recombinant plant expressed antibodies were extracted in 3 volumes of PBS (pH7.4) and purified by Ni-affinity chromatography [28]. 10 mg of ScFv (MW = 56 kDa) and the linker (succinimidyl-4-formylbenzamide) were dissolved in PBS. The linker solution was added to the ScFv solution under stirring, and the solution was agitated for 30 min in room temperature. The RVG peptide was synthesized by Pepscan (Lelystad, The Netherlands). 10 mg of the peptide (MW = 3 kDa) was dissolved in water and adjusted to pH7 with PBS. After the linker/ScFv solution was dialyzed in PBS for 15 min 4 times, it was added to the peptide solution under stirring at room temperature. After 2 h, the protein was dialyzed in PBS overnight. The reaction feed was 50% peptide and 50% ScFv, and the molar ratio was 18:1.

### 2.2 SDS-PAGE and western blot

Crude protein extracts from plant leaves were prepared 5 days after agro-infiltration and denatured by boiling in NuPAGE® LDS Sample Buffer. Proteins were separated on 4–12% gradient polyacrylamide gels (Life Technologies, UK). Proteins were visualised by Coomasie staining, or electrophoretically transferred to a nitrocellulose membrane for immunoblotting. Nitro-cellulose membranes were blocked (5% non-fat dried milk, 0.1% Tween20 in PBS) before being probed with horseradish peroxidase (HRP) conjugated mouse anti-E-tag antiserum (Abcam, UK) diluted at 1:5000 in 1% non-fat dried milk in PBST. Bands were visualised following addition of ECL plus detection reagent (GE Healthcare, UK).

### 2.3 Cells and viruses

Human embryonic kidney 293 cells expressing human α7-nicotinic acetylcholine receptor (HEKnAchR7) were reported previously [31]. The immortalized human brain capillary endothelial cell line (hCMEC/D3) [32] was purchased from Tebu Bio (France) and the cells were grown according to the manufacturer’s instruction. Silver Haired Bat rabies variant (SHBV) [33] was used for the rabies virus pathogenicity experiments.

### 2.4 nAchR binding and competition assay

HEK 293 cells or Neuroscreen-1 (Thermo-Fisher, UK) cells were seeded on 6-well plates. After 24 h, cells were placed on ice and incubated with ScFv or ScFv/RVG for 5 min (binding assay) or 30 min (entry assay). The cells were washed with PBS, then harvested into FACS tubes and incubated in cell fixation solution (BD Biosciences, USA) for 15 min. For the binding assay, samples were washed 3 times with 1% inactivated foetal calf serum (0.1% NaN_3)_ in PBS, pH 7.4. For the entry assay, samples were washed 3 times with permeabilization buffer (1% inactivated fetal calf serum, 0.1% NaN_3_, and 0.1% Saponin in PBS, pH 7.4) before the cells were incubated with 1:1000 mouse anti-E tag antiserum at 4 °C, overnight. The cells were then washed as before, before incubation with a goat anti-mouse IgG antiserum conjugated with cy5 (Jackson laboratory, USA) at 37 °C for 1 h. After further washing, the cells were resuspended in staining buffer and analysed by flow cytometry, using FACS CellQuest software (BD Biosciences, USA). For the competition assay, cells were pretreated on ice with either 2 × 10^7^ PFU of UV inactivated Rabies virus (CVS) [34] or 16 μM alpha bungarotoxin (Tocris Bioscience, UK) for 30 min, before the ScFv or ScFv/RVG conjugate was added. The binding and competition assays were analyzed in three independent experiments.

### 2.5 *In vitro* BBB transwell assay

An immortalized human brain capillary endothelial cell line (hCMEC/D3) was kindly provided by Prof. Pierre-Olivier Couraud (Institut Cochin, Université René Descartes, Paris, France) and Prof. Pierre-Emmanuel Ceccaldi (Institut Pasteur, Paris) [35]. Cells were seeded on the apical side of a Cultrex® Rat Collagen I (150 μg/ml; R&D Systems, USA) coated 0.9 cm^2^ polyethylene terephthalate filter insert with 3.0 μm porosity (BD Falcon, UK). The restrictive paracellular permeability of hCMEC/D3 cells was assessed by their low permeability to the non-permeant fluorescent marker Lucifer Yellow (LY) [29]. 10 µg of antibody preparation was added to the top chamber and the cells were incubated (37 °C; 5% CO_2_) and samples were taken after 2 h and 18 h to assess the media in the bottom chamber for the presence of antibody by virus neutralisation.

### 2.6 *In vivo* assessment of ScFV/RVG

All *in vivo* work was undertaken in BSL3/SAPO4 containment at the Animal and Plant Health Agency (APHA), following independent ethical review under strict Home Office guidelines (PPL70/7394). Molecules were administered to groups of mice by intraperitoneal inoculation. Intra-peritoneal administration (IP) of ScFv/RVG was compared to treatment with human rabies immunoglobulin (HRIG) as both a pre- and post-exposure treatment. Treatments with Favipiravir (T-705, a broad-spectrum RNA polymerase inhibitor), and T-705 with ScFv/RVG were also included in the study.

Mice (n = 12/group) received ScFv/RVG (40 IU/kg), HRIG (40 IU/kg), T-705 (300 mg/kg) or ScFv/RVG (40 IU/kg) + T-705 (300 mg/kg) or were controls receiving PBS following the same treatment schedule. Mice were tagged and numbered before using a random number generator to distribute mice into groups. Each group of 12 mice was randomly split across two boxes of 6 mice each, to take account of interactions among mice sharing boxes and any other differences between boxes. Groups of mice were treated for 10 consecutive days. The treatments were initiated either four hours before virus inoculation (−4hr), two days (+2d) after virus infection or 4 days (+4d) after virus inoculation. Virus used for inoculation was a bat rabies strain originally isolated from a human fatality following infection from an insectivorous bat [36]. Mice were challenged with 50 µl RABV at ∼105.8 TCID50/ml by intramuscular injection into the left hind leg. Mice were weighed daily during the 10 day treatment period to determine both weight loss due to infection and assign any possible adverse effect of treatment with ScFv, T705 or HRIG. Animals were monitored for 54 days and any deaths were recorded.

The data were analysed for treatment effects as a factorial design (5 treatments × 3 timings) by applying a multilevel mixed effects logistic regression to take account of potential correlation among mice in each box (melogit in Stata® 14, treating differences between boxes as random effects). Treatment effects were calculated as logits of mortality, where a logit is the logarithm of the odds ratio $\left(\text{logit}(p)={\text{log}}_{e}\left(\frac{p}{1-p}\right)\right)$. Treatments were compared using their logits: the treatment with higher logit results in higher mortality. A difference of zero indicates that two treatments have the same effect, a difference of 1.0 is equivalent to increasing mortality from 0.5 to 0.731, while a difference of −1.0 would be equivalent to reducing mortality from 0.731 to 0.5. The generalised linear statistical model assumed that the effect of combined treatments can be predicted by adding their effects on the logit scale. Deviation from this prediction indicates that the treatments interact. The model estimated standard errors for the differences between treatments, which allowed calculation of 95% confidence intervals and testing against a null hypothesis that the treatment effects were equal.

## 3 Results

### 3.1 Characterisation of the 62-71-3 ScFv and the ScFv/RVG conjugate

**Fig. 1:**
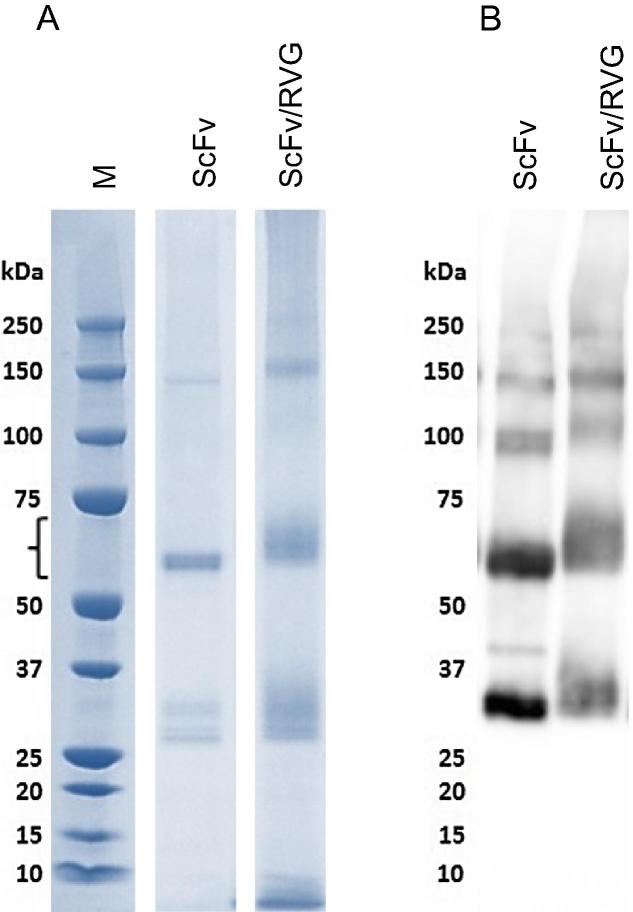
Characterisation of ScFv and ScFv/RVG conjugate. The plant-produced ScFv was purified by Ni affinity chromatography. The ScFv was chemically conjugated to chemically synthesized RVG peptide to produce ScFv/RVG. ScFv and ScFv/RVG conjugate were analysed by SDS-PAGE under reducing conditions, followed by (A) staining with Coomassie blue or (B) blotting onto nitrocellulose and probing with a mouse anti-E tag antiserum. (For interpretation of the references to colour in this figure legend, the reader is referred to the web version of this article.)

The purified ScFv and the ScFv/RVG conjugate were assessed by SDS-PAGE gel followed by Coomassie staining (Fig. 1A) or by immunoblotting with horseradish peroxidase conjugated mouse anti-E tag antiserum (Fig. 1B). A full size ScFv is detected predominantly at approximately 56 kDa, which was the major band detected. ScFv/RVG migrated slightly slower than ScFv as expected and the slight smearing of this band is consistent with variable levels of RVG peptide conjugation. Again, this band is the major component of the preparation. Higher molecular weight bands (approximately 150 kDa) are likely to represent ScFv aggregates, whilst lower molecular weight bands (30–35 kDa) are likely to represent ScFv degradation products. The identity of the bands was supported by western blot (Fig. 1B).

### 3.2 Neutralization of rabies virus

**Fig. 2:**
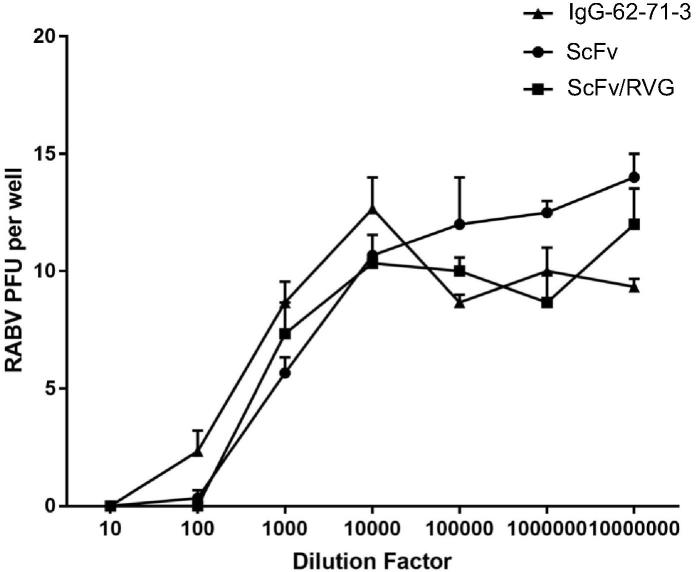
Rabies virus (ERA stain) neutralization by ScFv and ScFv/RVG conjugate compared with 62-71-3 mAb IgG antibody as assessed by RFFIT on BSR cells. Antibody starting concentrations were 0.5 mg/ml. Assays were performed in triplicate. Error bars indicate the SD.

The parent monoclonal antibody (62-71-3) and two versions of ScFv were tested to determine their capability to neutralize rabies virus (ERA strain) using a plaque-inhibition assay. The starting concentrations for all three antibodies was 0.5 mg/ml and the results suggest that the neutralizing activity of ScFv and ScFv/RVG conjugate was not significantly different to that of 62-71-3 mAb (Fig. 2).

### 3.3 Binding to nAchR and cell entry

**Fig. 3:**
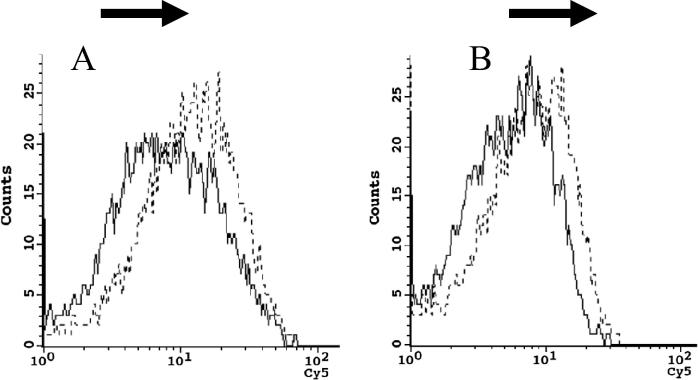
Binding and entry of 62-71-3 ScFv to 293 cells overexpressing nAchR by flow cytometry. Binding (A) and entry (B) were detected with mouse anti-E antiserum and cy5 conjugated goat anti-mouse IgG antiserum, Solid line: ScFv, Dotted line: ScFv/RVG conjugate. A representative result from triplicate experiments is shown.

The binding and penetration of ScFv and ScFv/RVG conjugate in HEK 293 cells overexpressing nAchR were tested by flow cytometry. A greater proportion of ScFv/RVG bound to the 293 cells as evidenced by the shift to the right of the dotted line compared to ScFv (solid line) (Fig. 3A). After a longer incubation (30 min) a greater amount of ScFv/RVG was associated with the 293 cells compared to ScFv (Fig. 3B), and this represents ScFv that has entered the target cells.

**Fig. 4:**
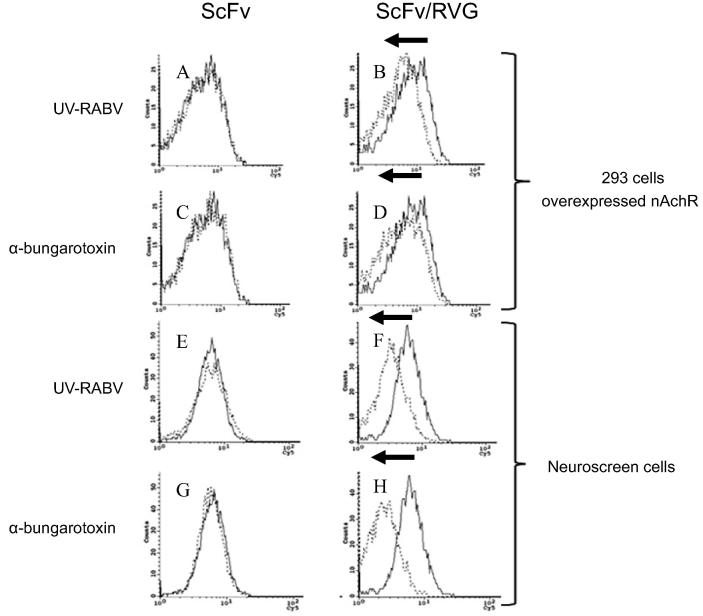
Inhibition of entry of ScFv/RVG conjugate into nAchR-overexpressing 293 cells and neuroscreen cells by irradiated rabies virus and α-bungarotoxin. Flow cytometry on nAchR-overexpressing 293 cells pre-treated with irradiated rabies virus (A, B) and α-bungarotoxin (C, D) before incubation with ScFv (A and C), and ScFv/RVG conjugate (B and D). Flow cytometry on neuronal 2a cells pre-treated with irradiated rabies virus (E, F) and α-bungarotoxin (G, H) before incubation with ScFv (E and G) and ScFv/RVG conjugate (F and H). Solid line: no inhibitor, Dotted line: pre-treated with irradiated rabies virus or α-bungarotoxin; A representative result from triplicate experiments is shown.

The specificity of binding between ScFv/RVG and HEK 293 cells via nAchR was tested by a competitive assay using irradiated rabies virus and α-bungarotoxin. The HEK 293 cell line was pre-incubated with each inhibitor, before incubation with ScFv or ScFv/RVG. No effect of either irradiated virus or α-bungarotoxin was observed in the case of ScFv (Fig. 4A and C, respectively). However, for ScFv/RVG there was a shift, with less ScFv/RVG detected within the cells in the presence of both inhibitors (Fig. 4B and D, respectively). The assays were repeated using Neuroscreen-1 cells, a model neuronal cell line, with identical results (Fig. 4E–H).

### 3.4 Passage of ScFv/RVG conjugate across an *in vitro* model of the blood brain barrier

**Fig. 5:**
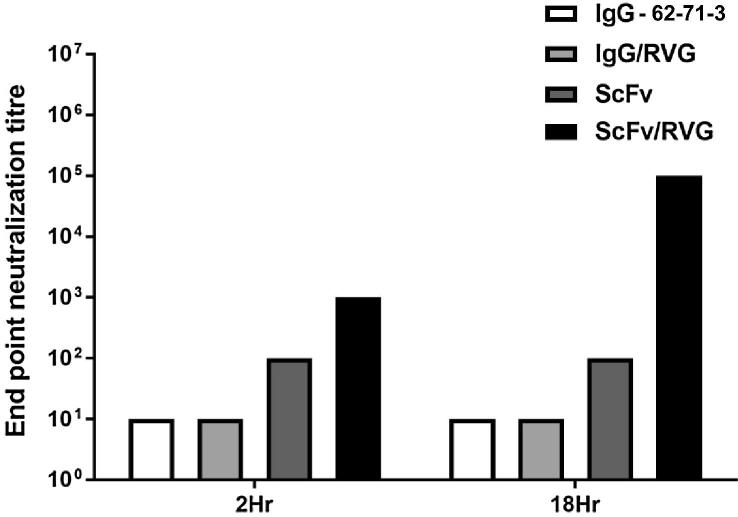
ScFv/RVG conjugate transports across *in vitro* BBB model. 10 μg antibodies were added to the upper chamber of hCMEC/D3 cells in the transwell. Media in the bottom well was tested for rabies virus neutralization assay after 2 and 18 h. A representative result from triplicate experiments is shown.

An *in vitro* BBB transport experiment was conducted on an hCMEC/D3 cell monolayer as described previously [35]. After addition of antibodies to the upper chamber, the media in the lower chamber was tested for rabies virus neutralizing activity after incubation periods of 2 and 18 h (Fig. 5). No evidence for the ability of full length 62-71-3 mAb to cross the cell monolayer was found. This is consistent with previous reports [28], [37] and demonstrates the integrity of the monolayer. Similarly, a 62-71-3 IgG/RVG conjugate was also unable to cross the monolayer. There was some detectable ScFv in the bottom chamber at both time points, but as the levels were similar at both time points, we interpret this to represent slight leakage of the monolayer to small proteins. In contrast, a greater amount of ScFv/RVG passed through the hCMEC/D3 cells, and the concentration of ScFv/RVG as measured by virus neutralising activity of the media in the bottom well increased approximately 100-fold after 18hr incubation (Fig. 5).

### 3.5 *In vivo* assessment of ScFv/RVG

**Fig. 6:**
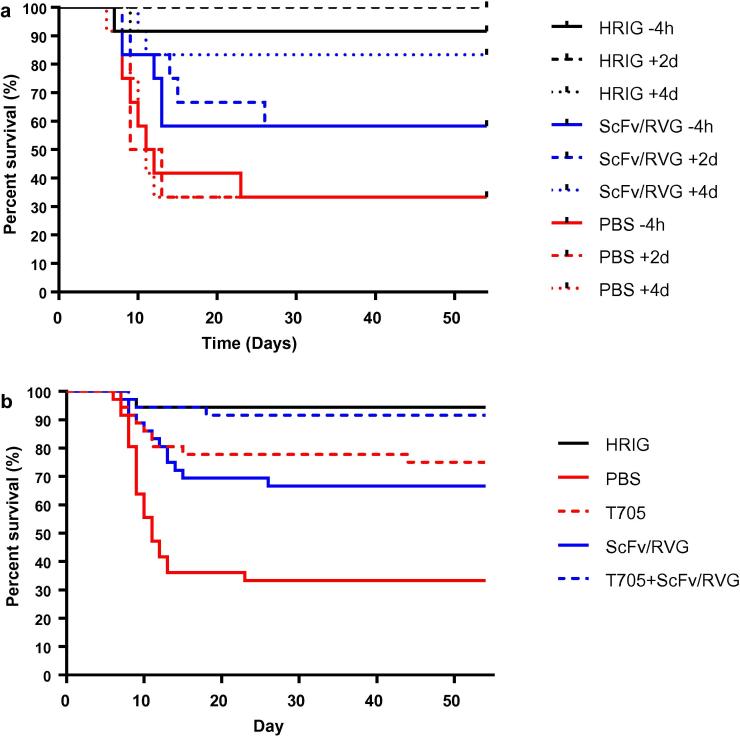
(a) Mouse survival curves for the three treatments ScFv/RVG, HRIG and PBS only controls at three different time points following inoculation with rabies virus. (b) Mouse survival curves for the five treatments ScFv/RVG, T-705, ScFv/RVG with T-705, HRIG and PBS only controls, combining observations across three different timings, which did not significantly affect treatment effects.

**Fig. 7:**
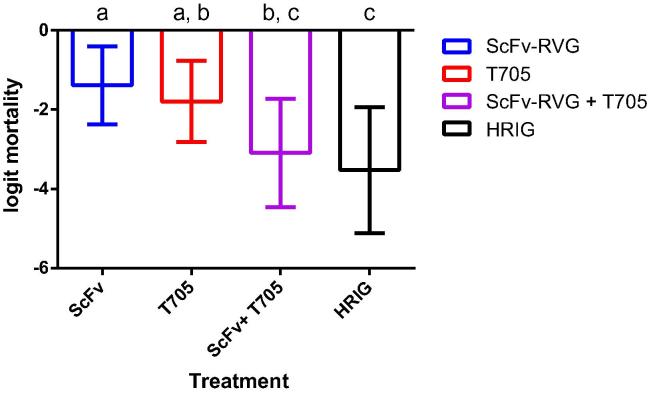
Logit mortality for four treatments ScFv/RVG, T-705, ScFv/RVG with T-705 and HRIG relative to PBS only controls, assuming treatment effects did not interact with timing. Bars show 95% confidence intervals estimated from a mixed effects logistic regression. Letters above the bars group treatments with similar mortality; treatments differ significantly at the 95% confidence level if they do not share any matching letters.

The effectiveness of the ScFv/RVG conjugate against rabies viral challenge was assessed *in vivo*. There was a clear trend showing greatest mortality in PBS treated groups, compared with those treated with HRIG (lowest) and ScFv/RVG (Figs. 6, 7). Unexpectedly, even 4 days after viral challenge, HRIG was almost totally protective, and there was no evidence of any effect from the timing of treatments. Among the four treatments ScFv/RVG, T-705, ScFv/RVG with T-705 and HRIG, the estimated effect on logit mortality from treating at 2d relative to −4h = 0 (95% confidence interval −1.08 to 1.08); 4d relative to −4h = −0.16 (−1.26 to 0.94). T705 reduced mortality to a similar degree compared with ScFv/RVG (Figs. 6B, 7), and the group treated with the combination of ScFv/RVG conjugate with T-705 reduced mortality to a level similar to HRIG (Fig. 6B, 7). However, although the best model of the experimental observations was that the effects of T705 and ScFv/RVG were additive, the difference between T705 alone and the combination of T705 with ScFv/RVG conjugate did not reach the threshold for statistical significance at P < 0.05 (Fig. 7).

## 4 Discussion

The blood-brain barrier remains a major bottleneck for drug development, for rabies and many other brain diseases. Several strategies have been developed, including the use of nanotechnology employing liposomes [38], polymeric nanoparticles [39], micelles [40], gold particles [41], etc. Another strategy is the use of antibodies to target receptors on the surface of endothelial cells allowing transport of drugs into the brain. Examples include antibodies against the transferrin receptor [42], [43], [44], insulin receptor [45], [46] or the low density lipoprotein receptor [47]. Peptides have also gained attention for their potential to mediate delivery across the BBB [48], [49], [50]. The rabies virus glycoprotein (RVG) peptide used in this study binds specifically to the acetylcholine receptor (nAchR) expressed on neuronal cells. Several studies have demonstrated that RVG peptide can deliver siRNA [21] and proteins [22], [51] through the BBB.

Our previous work demonstrated expression of a ScFv version of the rabies neutralising monoclonal antibody 62-71-3 *in planta* [27]. The lyssavirus neutralisation activity of the ScFv was equivalent to that of the IgG parent antibody. In a preliminary study, an ScFv-RVG fusion protein was engineered, and we were able to demonstrate some of the functional characteristics of this molecule [28]. However, the expression level of this molecule in plants was extremely low, approximately 2 mg/kg fresh leaf weight, which is significantly below the level required for commercial viability. By comparison, IgG antibodies are currently being developed that express in *Nicotiana* in the range of 100 mg/kg fresh leaf weight [52].

In this study, our strategy was to express the 62-71-3 ScFv molecule separately in *Nicotiana benthamiana* and following purification, use chemical conjugation to synthetic RVG peptide. The ScFv was expressed at 35–50 mg/kg fresh leaf weight which has important advantages in terms of downstream processing and purification, and consequently on commercial viability. Chemical conjugation of RVG peptide to ScFv is also potentially advantageous because multiple peptides could be attached to a single ScFv molecule, thereby increasing affinity for the nAchR. Indeed, as shown in the SDS-PAGE and western blot of the ScFv/RVG conjugate, the product band indicates molecules with a range of sizes.

Importantly, RVG conjugation did not affect rabies neutralisation activity, and there was no discernible difference between unconjugated ScFv and ScFv/RVG. The ScFv/RVG conjugate did mediate binding and entry into cells overexpressing nAchR and a neuron-like cell line (neuroscreen cells) and the role of nAchR in this interaction was demonstrated by the ability of both rabies virus and alpha-bungarotoxin to competitively inhibit ScFv. Alpha-bungarotoxin is a neurotoxin that binds nAchR at the same site as rabies glycoprotein [53].

An *in vitro* model was utilised to investigate the potential transport of different antibody based molecules across the blood brain barrier. This model was impermeable to the full length 62-71-3 IgG mAb as expected. Conjugating RVG to 62-71-3 IgG made no difference, indicating that that the size of IgG is a limiting factor. Although there was some apparent passage of ScFv across the BBB model, this was significantly enhanced in the case of ScFv/RVG. The increasing concentration of neutralising activity in the lower chamber of this assay with time, in comparison with the result using unconjugated ScFv alone, suggests that transport was mediated by an active mechanism.

An *in vivo* assessment of ScFv/RVG was subsequently attempted using a murine model of rabies virus infection and different treatment schedules with either HRIG or the ScFv molecule. For this experiment, treatment schedules were designed on the hypothesis that at 4 h before inoculation and 2 days post inoculation, the infecting virus would still be in the periphery and that an established neuronal infection had not yet been initiated. The 4 day post inoculation treatment schedule was chosen because it was expected that an infection of the central nervous system would have established, so it should be possible to demonstrate protective efficacy from ScFv/RVG due to greater accessibility to the brain [54].

However, the results suggest that the virus took longer to reach the CNS than expected. HRIG was protective when delivered at all time points, even though it is well established that HRIG does not provide protection once rabies virus infection enters the CNS. So unfortunately, no conclusions can be drawn regarding potential ScFv mediated protection within the CNS. With no significant effect from the timing of treatments, ScFv/RVG halved mortality relative to the control treatment, but did not match the 90% protection observed for HRIG. Although the dosages administered were equivalent in terms of International Units/kg, ScFv/RVG performed less effectively than HRIG. This is likely to be due to different pharmacokinetics, as without Fc, ScFv/RVG would be expected to have a shorter serum half life [55]. Favipiravir (T705) performed similarly to ScFv/RVG. However, the combination of ScFv/RVG with T-705 appeared to match the protection from HRIG, most likely because the effects of ScFv/FVG and T-705 were additive, but the evidence is not decisive. The relative performance of ScFv/RVG and HRIG when treatment is sufficiently delayed for mortality to be high with HRIG treatment remains unknown. This study did however, confirm the protective property of ScFv/RVG *in vivo*, and demonstrates that the chemical conjugation process does not affect the viral neutralisation properties of the ScFv *in vivo.* A definitive pre-clinical study demonstrating protective efficacy in a robust model for central nervous system infection by rabies virus is now required.

In conclusion, the adaptation of ScFv through conjugation to a 29 amino acid RVG peptide has enabled greater bioavailability of the molecule. In particular, the approach adopted in this study overcomes the problem of low yield, and the scalable production of rabies ScFv molecule in plants is promising. RVG peptide synthesis and the conjugation process are readily available commercially and available under Good Manufacturing Practice when necessary. This leads to the possibility for rapid large scale production of the conjugated molecule and relatively quick translation to clinical trial. The development and clinical evaluation of new tools for post exposure control for rabies virus infection in endemic areas is a matter of some urgency.
